# Hereditary sensory neuropathy type I

**DOI:** 10.1186/1750-1172-3-7

**Published:** 2008-03-18

**Authors:** Michaela Auer-Grumbach

**Affiliations:** 1 Institute of Human Genetics, Graz, Austria; 2 Department of Internal Medicine, Medical University of Graz, Austria

## Abstract

Hereditary sensory neuropathy type I (HSN I) is a slowly progressive neurological disorder characterised by prominent predominantly distal sensory loss, autonomic disturbances, autosomal dominant inheritance, and juvenile or adulthood disease onset. The exact prevalence is unknown, but is estimated as very low. Disease onset varies between the 2^nd ^and 5^th ^decade of life. The main clinical feature of HSN I is the reduction of sensation sense mainly distributed to the distal parts of the upper and lower limbs. Variable distal muscle weakness and wasting, and chronic skin ulcers are characteristic. Autonomic features (usually sweating disturbances) are invariably observed. Serious and common complications are spontaneous fractures, osteomyelitis and necrosis, as well as neuropathic arthropathy which may even necessitate amputations. Some patients suffer from severe pain attacks. Hypacusis or deafness, or cough and gastrooesophageal reflux have been observed in rare cases. HSN I is a genetically heterogenous condition with three loci and mutations in two genes (*SPTLC1 *and *RAB7*) identified so far. Diagnosis is based on the clinical observation and is supported by a family history. Nerve conduction studies confirm a sensory and motor neuropathy predominantly affecting the lower limbs. Radiological studies, including magnetic resonance imaging, are useful when bone infections or necrosis are suspected. Definitive diagnosis is based on the detection of mutations by direct sequencing of the *SPTLC1 *and *RAB7 *genes. Correct clinical assessment and genetic confirmation of the diagnosis are important for appropriate genetic counselling and prognosis. Differential diagnosis includes the other hereditary sensory and autonomic neuropathies (HSAN), especially HSAN II, as well as diabetic foot syndrome, alcoholic neuropathy, neuropathies caused by other neurotoxins/drugs, immune mediated neuropathy, amyloidosis, spinal cord diseases, tabes dorsalis, lepra neuropathy, or decaying skin tumours like amelanotic melanoma. Management of HSN I follows the guidelines given for diabetic foot care (removal of pressure to the ulcer and eradication of infection, followed by the use of specific protective footwear) and starts with early and accurate counselling of patients about risk factors for developing foot ulcerations. The disorder is slowly progressive and does not influence life expectancy but is often severely disabling after a long duration of the disease.

## Disease name, synonyms, and historical terms (*)

Hereditary sensory neuropathy type I (HSN I)

Hereditary sensory and autonomic neuropathy type I (HSAN I)

Charcot-Marie Tooth type 2B syndrome (HMSN 2B)

Hereditary sensory radicular neuropathy*

Ulcero-mutilating neuropathy*

Thevenard syndrome*

Familial trophoneurosis*

Mal perforant du pied*

Familial syringomyelia*

## Definition

Hereditary sensory neuropathy type I (HSN I) is a slowly progressive neurological disorder characterised by prominent predominantly distal sensory loss, autonomic disturbances, autosomal dominant inheritance, and juvenile or adulthood disease onset.

## Epidemiology

Hereditary sensory neuropathy type I (HSN I) constitutes a clinically and genetically heterogenous group of disorders of low prevalence. No detailed epidemiological data are currently available. The disease frequency is still reflected by reports of several affected families. Although the impressive clinical features of HSN I are seen by neurologists, general practitioners, orthopaedists and dermatologists, the condition might still be under-recognised. This is particularly true for cases without family history and those HSN I patients who do not exhibit the characteristic clinical features.

## Clinical description

Hereditary sensory neuropathies (HSN), also known as hereditary sensory and autonomic neuropathies (HSAN), belong to the large group of hereditary neuropathies. Typically, the HSNs predominantly affect peripheral sensory and autonomic neurons but there is also variable motor involvement [1]. The classification of the HSNs proposed by Dyck *et al *was suggested before the detection of responsible genes and was thus based on the age at onset, the mode of inheritance and the predominant phenotype. It comprises five main subtypes (HSN, HSAN types 1–5). HSN (HSAN) type I (HSN I) is characterised by autosomal dominant inheritance and juvenile or adulthood disease onset. The congenital and early onset forms of HSN are subcategorised as subtypes HSN II – V and are transmitted by an autosomal recessive trait [1].

This review focuses on HSN type I. Molecular genetic studies in the past years have demonstrated genetic heterogeneity between the HSN I subtypes. The summary provided in the next paragraph describes clinical and neurological abnormalities in which the diagnosis HSN I should be considered in a patient or a family.

A main and consistent feature of HSN I is the reduction of sensation sense which is mainly distributed to the distal parts of the upper and lower limbs (Table 1). Disease onset varies between the 2^nd ^and 5^th ^decade of life. At the beginning patients often notice distal sensory loss and/or slow healing of wounds and/or chronic skin ulcers. The latter changes appear due to permanent pressure, *i.e*. long walks or when patients wear shoes that do not fit well. Minimal wounds or blisters may then lead to deep foot ulcerations. Other patients first recognise that they cannot distinguish warm and cold stimuli, and that they are insensitive to pain. When they get painless burns and injuries they seek medical advice [1]. Neurological examination detects distal sensory disturbances, which may affect all sensory qualities or selectively affect pain and temperature sensation with preservation of vibration and joint position sense. This dissociated sensory involvement is typically seen in patiens with *SPTLC1 *mutations and occurs in early stages of the disease [2]. The degree of motor involvement is highly variable, even within families, and ranges from absent to severe distal muscle weakness and wasting of foot extensors leading to steppage gait. In patients with marked involvement of the motor nerves, the additional presence of prominent sensory abnormalities and foot ulcerations are the only signs to separate HSN I from hereditary motor and sensory neuropathy (HMSN, *i.e*. Charcot-Marie-Tooth syndrome, CMT) [3,4]. Foot deformity promotes ulcerations and skin changes such as hyperkeratosis at pressure points, which is also often prominent in HSN I. Serious and common complications are spontaneous fractures, osteomyelitis and necrosis, and neuropathic arthropathy which may even necessitate amputations. Also autonomic features are often observed and usually consist of sweating disturbances. With progression of the disease, loss of sensation and motor weakness may spread from distal parts of the lower limbs to more proximal parts, but also the hands may become similarly involved. In some reported HSN I families, hypacusis or deafness, or cough and gastrooesophageal reflux have been observed. Some patients experience positive symptoms of severe shooting, burning and lancinating pain in the limbs or even in the trunk [1]. These symptoms are more frequently described in patients with *SPLTC1 *mutations [2]. The disorder is slowly progressive, but is often severely disabling after a long duration of the disease [1,2,5].

**Table 1:** Primary diagnostic criteria

**Main features**
Prominent/predominant distal sensory loss
Repeated foot ulcerations/acromutilations
Osteonecrosis

**Additional criteria**

Variable distal motor involvement
Foot deformity
Autonomic disturbances
Skin changes (hyperkeratosis, blisters, onychomycosis, etc.)
Autosomal dominant inheritance

## Electrophysiological and nerve biopsy findings

In HSN I there is broad variability of electrophysiological abnormalities within and between families. Primarily axonal nerve damage of both motor and sensory nerves has been shown. Sensory potentials are usually absent in the lower limbs but are often recordable or even normal in the upper limbs, particularly in females [1,6]. The study of Whitaker *et al *in the family later shown to carry a *SPTLC1 *mutation and the study by Dubourg *et al *also show motor conduction slowing, possibly implying a demyelinating process [7,8]. Neurophysiological studies in CMT2B patients with a *RAB7 *mutation showed a mixed motor and sensory neuropathy with axonal and sometimes also demyelinating nerve damage [2,9]. Sural nerve biopsy findings were well studied in six English patients with a *SPTLC1 *mutation. In severely affected nerves only a very few myelinated fibres remained but electron microscopy showed a reasonable number of unmyelinated axons although the presence of stacks of flattened Schwann cell processes suggested unmyelinated axon loss. There was also some evidence for primary demyelination [6].

## Aetiology

HSN I is a genetically heterogenous condition. In the past two decades the introduction of genome-wide linkage studies enabled genetic testing of several HSN families and resulted in the elucidation of the molecular genetic background of these diseases. Detailed family studies clearly demonstrated clinical and genetic heterogeneity of HSN I and three gene loci and two genes were subsequently identified [10]. Table 2 summarises the known genes and gene loci and the phenotypic characteristics of the HSN I subtypes. A clinician should always be aware that the diagnosis of HSN I depends on patients having severe sensory loss sufficient to cause painless injuries or ulcerations. A comprehensive family history may also often serve to a correct diagnosis.

**Table 2:** Classification of hereditary sensory neuropathies type I (HSN I)

***Subtype***	***Hallmark/additional features***	***Locus***	***Gene***	***OMIM #***
HSN IA	Predominant loss of pain and temperature sensation, sometimes initial sign with long preservation of vibration sense, burning and lancinating pain, variable distal motor involvement, which may be severe.	9q22.1–q22.3	*SPTLC1*	162400
HSN IB	Predominant sensory neuropathy with cough and gastro-oesophageal reflux, rarely foot ulcerations; normal distal muscle strength.	3p24–p22	unknown	608088
HSN IC (= CMT2B, HMSN IIB)	Prominent distal motor involvement, often as initial sign of the disease, sensory loss of all qualities, acro-mutilating complications.	3q21	*RAB7*	600882
HSN ID	Prominent sensory loss and mutilations in hands and feet, acropathy; variable motor involvement.	unknown	unknown	-

A subdivision into HSN IA, HSN IB, HSN IC, and HSN ID which is based on the different genetic background is proposed here.

### 1. Hereditary sensory neuropathy type IA (HSN IA), also known as hereditary sensory and autonomic neuropathy (HSAN) type I

Historically, several families with juvenile and adulthood HSN and suggestive autosomal dominant inheritance have been reported. Most of the patients described in these early reports exhibited the typical features of HSN with pronounced distal sensory disturbances and acro-mutilations, and sometimes also distal muscle involvement. These families were diagnosed as HSN I in the classification proposed by Dyck [1]. In 1996, Nicholson *et al *re-examined family members of a previously reported typical HSN I kindred [11]. Blood samples were taken from affected and unaffected family members for genetic linkage analysis. Subsequently, the disease gene in this HSN I family was linked to chromosome 9q21–q22 and this locus was confirmed in two further Australian families [12]. In 2001, Dawkins *et al *showed that specific missense mutations in the human gene serine palmitoyltransferase long chain (*SPTLC1*) lead to HSN I. In this study, 11 out of 24 families with HSN were screened and carried a pathogenic mutation in the *SPTLC1 *gene. This gene is ubiquitously expressed and comprises 15 exons [13]. The most common mutation was a single base substitution c.399T > G in exon 5 of the *SPTLC1 *coding region resulting in a single amino acid substitution of cysteine to tryptophan (C133W) and was found in eight HSN I families of Australian, English and Canadian origin. Two further less common missense mutations in exons 5 and 6 (c.398G > A and c.431T > A, *i.e*. C133Y and V144D) were identified in an Austrian family and in a family of Australian/English descendent [13]. Independently, Dawkins' findings were confirmed by Bejaoui *et al *who reported the C133Y and the C133W mutations in two further unrelated families with typical HSN I [14]. Lateron, a novel missense mutation c.1160G > C (G387A) in exon 13 was identified in twin sisters from Belgium [15]. Known *SPTLC1 *mutations were also confirmed in HSN I families from Czechia and Portugal [16,17]. Nicholson *et al *pointed out that the sensory-motor neuropathy phenotype caused by the c.399T > G point mutation was the same as that reported by Campell and Hoffmann and, possibly, the same as that originally described by Hicks. Haplotype analysis in the families reported by Dawkins demonstrated that three Australian families of English extraction and three English families had the same haplotype on chromosome 9 suggesting a common founder as was the case in six of eight families in an English study [6,18]. Subsequently, it was suggested that *SPTLC1 *might be the causative gene in the majority of HSN I patients. However, this was questioned by a large study by Klein *et al*, in which only one *SPTLC1 *mutation was identified 25 HSN families tested [19]. All mutations which have been identified as a cause of HSN I are summarised in the Mutation Database of Inherited Peripheral Neuropathies [20].

Serine palmitoyltransferase (SPT) is a pyridoxal-5'-phosphate dependent enzyme, which is suggested to be a key enzyme for the regulation of sphingolipid levels in cells. SPT in humans consists of two hetero subunits, SPTLC1 and SPTLC2 (or LCB1 and LCB2 in mammals), which are both bound to the endoplasmic reticulum (ER) [21]. Regulation of sphingolipid synthesis at the SPT step prevents a harmful accumulation of metabolic sphingolipid-intermediates including sphingoid bases and ceramide, whereas repression of other anabolic steps in the spingolipid synthetic pathway may cause intermediates to accumulate. It is still unclear why mutations in a protein widely expressed in all tissues, trigger pathology that is highly restricted to specific subsets of cells within a tissue [22]. The C133W and V144D *SPTLC1 *mutations were originally suggested to increase the serine palmitoyltransferase function with higher levels of glycosyl ceramide compared to controls behaving as gain of function mutations [13]. However, more recent studies showed that both mutations reduce the normal serine palmitoyltransferase activity in various mammalian cell types, including cultured lymphoblasts from HSN1 patients indicating that *SPTLC1 *mutations are dominant inactivating [23,24]. The reason for the discrepancy of the two studies is unknown [21]. Recently, Dedov *et al *carried out functional studies in order to detect mechanisms leading to the HSN I phenotype. Their tests using cells from HSN I patients with the mutation T399G-(Cys133Trp) in the *SPTLC1 *gene revealed a reduction of SPT activity in transformed lymphocytes of 44%. Interestingly, this had no effect on various spingolipid associated functions as *de novo *biosynthesis, cellular sphingolipid content, cell proliferation or death (apoptosis and necrosis). Other tests showed similar results with no effects on viability of cells after removal of extracellular sphingolipids, on permeability to triton X-100 of primary lymphocytes, on viability or in whole blood counts. Thus, the authors concluded a sufficient actitivity of the non mutant allele for adequate sphingolipid biosynthesis and cell viability. The authors speculated that neurodegeneration in HSN1 is due to rather subtle and long term effects like abnormal protein(s) similar to other neurodegenerations.

*SPTLC2 *is the second gene for the SPT protein, is located on the chromosome 14q24.3–q31 and comprises 12 exons [21]. Screening of 12 index patients from families with HSN excluded for mutations in the *SPTLC1 *gene did not reveal any pathogenic mutations in the *SPTLC2 *gene. The authors therefore concluded that *SPTLC2 *mutations are not a common cause for hereditary sensory neuropathy [25].

### 2. Hereditary sensory neuropathy type IB (HSN IB) (also known as hereditary sensory neuropathy with cough and gastrooesophageal reflux)

In 2002, Spring *et al *reported a family with an autosomal dominant hereditary HSN. Patients had distal sensory loss usually without foot ulcerations but adult onset of gastro-oesophageal reflux and cough and no motor symptoms. Cough could be triggered by noxious odours and could lead to syncope. Nerve conduction studies, and sural and skin biopsies revealed a sensory axonal neuropathy. Audiometry showed sensorineural hearing loss in 4 out of 10 affected individuals [26]. The disease locus in this family was linked to a 3.42 cM interval on chromosome 3p22–p24 in 2003, and was also confirmed in a second family with a similar phenotype [27]. Since then no further families with this rare form of HSN have been described. The gene involved in this disease still remains to be identified.

### 3. Hereditary sensory neuropathy type IC (HSN IC), also known as Charcot-Marie-Tooth syndrome type 2B (CMT2B)

In 1995, Kwon *et al *carried out a clinical and genetical study in a large American family with autosomal dominant HMSN 2 (CMT2) [3]. In this family affected individuals showed prominent distal muscle weakness and wasting, sensory loss, and foot ulcerations which were frequently complicated by toe and/or foot amputations. A whole genome scan demonstrated linkage to chromosome 3q13–q22 [3]. Due to the prominent involvement of motor fibres the disorder of this family was genetically subcategorised among the hereditary motor and sensory neuropathies (HMSN) and was termed HMSN2B (CMT2B) [3,4]. With regard to the prominent ulcero-mutilating complications this classification was questioned from the beginning because some authors argued that the disease should have better been called HSN type 1 [28]. The disease locus on chromosome 3q13–q22 was confirmed in a small Scottish family and subsequenly in an Austrian kindred and the critical interval was considerably refined [9,29]. Finally in 2003, the causative gene of CMT2B was identified and two missense mutations in the small GTPase late endosomal protein *RAB7 *were detected [30]. The Val162Met was found in exon 4 in the previously reported American and Scottish families as well as in a small Austrian family. In the large Austrian family described in 2001, the Leu129Phe in exon 3 was found to be related to the disease. Later, this mutation was also confirmed in further small Austrian families suggesting a common founder, and in patients from Belgium and Czechia [17,30]. A third missense mutation in exon 4 of the *RAB7 *gene (Asn161Thr) was reported in 2004 in an English family [31]. The mutation is located in a highly conserved region adjacent to the reported Val162Met mutation, and thus suggests that this region might be a functionally important hotspot for *RAB7 *mutations [32].

*RAB7 *consists of 5 exons and belongs to the Rab family of Ras-related GTP-ases. The Rab proteins are essential for the regulation of intracellular membrane trafficking. They may have a role in linking vesicles and target membranes to the cytoskeleton [32,33]. The *RAB7 *gene has been localised to late endosomes and has been shown to be important in the late endocytic pathway. Although the function of *RAB7 *has already been studied in detail, it remains still unknown how mutations in the ubiquitously expressed *RAB7 *gene cause a CMT2B neuropathy [30].

### 4. Hereditary sensory neuropathy type 1D (HSN ID)

Further genetic heterogeneity has been suggested in HSN I [19,34,35]. Klein *et al *could not identify any mutations in the *RAB7 *gene in the large series of HSN families and in sporadic HSN patients of whom several affected individuals also had marked peroneal muscle wasting. Also, linkage to the known HSN I loci has been excluded in a few further families with autosmal dominant inheritance. Thus, further genetic linkage studies in large families are needed to identify new causative genes of HNS I [19].

## Diagnostic methods

In a single patient, the clinical diagnosis of HSN I is based on the observation of signs and symptoms described above, and is supported by a family history suggesting autosomal dominant inheritance. The diagnosis is supported by ancillary tests such as nerve conduction studies which are needed to confirm a sensory and motor neuropathy predominantly affecting the lower limbs. In sporadic cases acquired neuropathies have to be excluded (see differential diagnosis) by use of several laboratory tests. Radiological studies, including magnetic resonance imaging, are useful when bone infections or necrosis are suspected. With the discovery of several distinct HSN loci, and ultimately the deciphering of the underlying gene defects, the definitive diagnosis is now molecular and is based on the detection of mutations by direct sequencing of the *SPTLC1 *and *RAB7 *genes. Large families in which mutations in *SPTLC1 *and *RAB7 *are excluded can be used for genome wide linkage studies to detect a novel HSN I locus or to confirm linkage to the third known HSN I locus (*i.e*. the HSN IB locus). Such large HSN families will be most helpful to identify further genes which are involved in the pathogenesis of HSN I.

## Differential diagnosis

HSN I must be distinguished from other forms of hereditary sensory neuropathies (HSN types II-V). The phenotype of HSN II is often similar to that of HSN I. The main differences are the autosomal recessive pattern of inheritance of HSN II, an earlier disease onset, diffuse sensory loss which is sometimes distributed to the whole body, and less or no motor symptoms in HSN II. HSN II patients also often exhibit early-onset of severe acro-mutilations in the fingers. HSN III to HSN V can be easily distinguished from HSN I because of congenital disease onset. Also, these subtypes exhibit typical features such as the predominant autonomic disturbances in HSN III or congenital loss of pain and anhidrosis in HSN IV.

Particularly in sporadic patients with an HSN I phenotype it is important to exclude acquired causes of ulcero-mutilating neuropathies. The major differential diagnoses are the diabetic foot syndrome and alcoholic neuropathy. Exclusion of other causes of neuropathies can usually be done by laboratory and radiological studies, and requires interdisciplinary discussion between neurologists, dermatologists, and orthopaedics. Table 3 summarises other diseases which may mimick HSN I or are associated with plantar foot ulcers.

**Table 3:** Differential diagnosis of hereditary sensory neuropathy type 1 and foot ulcerations

Hereditary sensory neuropathy type II
Diabetic foot syndrome
Alcoholic neuropathy
Neuropathies caused by other neurotoxins/drugs
Immune mediated neuropathy
Amyloidosis
Spinal cord diseases
Tabes dorsalis
Lepra neuropathy
Amelanotic melanoma and other skin tumours
Artefacts

## Genetic counselling

As the disorder is inherited by an autosomal dominant trait, previous family history is often reported. There is thus a Mendelian risk of 50% for subsequent generations independently from the sex of the affected individual and the child. Genetic counselling is an important tool for preventing new cases if this is wished by at-risk family members. Predictive testing of mutation carriers necessitates accurate genetic counselling but is certainly useful for young people to avoid serious complications of the disease.

## Prenatal diagnosis

Molecular genetic diagnosis can be considered in families where the disease causing mutation is known. However, termination of pregnancy is not recommened in HSN1. When a patient is male, artificial insemination with donor sperm is another option that could be discussed with the couple during genetic counselling.

## Management

No gene-based therapies are available to date for any variant of autosomal dominant HSN. Yet, accurate diagnosis is important and is requested by patients and at-risk family members. Ulcero-mutilating complications are the most serious, prominent and leading diagnostic feature in HSN I. The painless neuropathic foot ulcerations observed in several subtypes of autosomal dominant HSN often mimic foot ulcers caused by diabetic neuropathy and thus resemble a "*pseudodiabetic foot syndrome"*. Therefore, treatment of foot ulcers and infections can follow the guidelines given for diabetic foot care which starts with early and accurate counselling of patients about risk factors for developing foot ulcerations. This of course includes orthopaedic care and use of well fitting shoes without pressure points. To date, treatment of foot complications has reached an efficient level allowing treatment on an outpatient basis. Early treatment of foot complications often avoids hospitalisation and, in particular, complications like amputations. Principles of therapy are removal of pressure to the ulcer, eradication of infection and specific protective footwear afterwards.

## Prognosis

If patients with HSN I receive appropriate counselling and treatment, the prognosis is good. Early treatment of foot infections may avoid serious complications. Also the complications are manageable, allowing an acceptable quality of life. The disease is slowly progressive and does not influence the life expectancy.

## Unresolved questions

It is still unclear how mutations in the *SPTLC1 *and *RAB7 *genes lead to the phenotype of HSN I. For many families and single HSN cases the genetic background still remains unknown due to the genetic heterogeneity of the autosomal dominant HSN. There is still no causative treatment of HSN. Pharmacological treatment or gene therapy are needed but require a better understanding of the molecular and functional mechanisms underlying the different genetic subtypes of HSN I.
